# Do the omeprazole family compounds exert a protective effect against influenza-like illness?

**DOI:** 10.1186/1471-2334-14-297

**Published:** 2014-06-02

**Authors:** Roberto Gasparini, Piero Luigi Lai, Francesca Casabona, Cecilia Trucchi, Sara Boccalini, Maria Luisa Cristina, Stefania Rossi, Daniela Amicizia, Donatella Panatto

**Affiliations:** 1 Department of Health Sciences, Genoa University, Genoa, Italy; 2 Inter-University Centre for Research on Influenza and other Transmitted Infections (CIRI-IT), Genoa, Italy; 3 Department of Health Sciences, Florence University, Firenze, Italy; 4 Department of Molecular and Developmental Medicine, Siena University, Siena, Italy

**Keywords:** Influenza-like illness (ILI), Omeprazole family compounds, Epidemiology, Prevention

## Abstract

**Background:**

Infections by influenza viruses place a heavy burden on public health and economies worldwide. Although vaccines are the best weapons against influenza, antiviral drugs could offer an opportunity to alleviate the burden of influenza. Since omeprazole family compounds block the “proton pump”, we hypothesized that they could interfere with the mechanism of fusion of the virus envelope and endosomal membrane, thereby hindering the M2 proton pump mechanism of influenza viruses.

**Methods:**

A matched case-control study was performed in 2010-2011 in Italy. Cases were subjects aged over 18 years with a diagnosis of Influenza-like Illness (ILI); 254 case-control pairs were recruited. A multivariable conditional logistic regression analysis was used to assess the association between the prevention of ILI and the administration of omeprazole family compounds. The interaction between omeprazole family compounds and influenza vaccination was also examined.

**Results:**

After control for potential confounders, subjects treated with omeprazole family compounds displayed a lower risk of catching ILI (OR_adj_ = 0.29, 95% CI: 0.15-0.52). The risk of ILI in unvaccinated non-OFC users was about six times than that in vaccinated OFC users.

**Conclusions:**

Although confirmation is necessary, these results suggest that omeprazole family compounds could be profitably used in the prevention of ILI.

## Background

Infections by influenza viruses place a heavy burden on public health and economies worldwide. The World Health Organisation (WHO) has estimated that 1 billion cases of illness, 3-5 million complicated cases and 300,000 – 500,000 deaths occur worldwide every year [1]. In the United States, more than 200,000 hospitalizations per year for respiratory and heart conditions have been attributed to seasonal influenza [2]. In Italy, considering seasonal epidemic periods alone, it has been estimated that 25 million cases of Influenza-like Illness (ILI) occurred from 1999 to 2008, with an average of 2.5 million cases per year [3].

Molinari et al. estimated that, on the basis of the US population in 2003, annual influenza epidemics resulted in an average of 610,660 life-years lost, 3.1 million days of hospitalization and 31.4 million outpatient visits. These authors also estimated that, of the 31.4 million outpatient visits, 9.6 and 14.3 million involved subjects aged 18-64 years and over 65 years, respectively [4].

Vaccines are the principal weapons against influenza. However, because it takes time to produce an antigenically appropriate immunogenic vaccine and to deliver it to populations, especially in the event of a pandemic, antiviral drugs could offer a good opportunity to alleviate the burden of seasonal and pandemic influenza [5]. To date, there are 4 main anti-influenza medications: two amantadanes (amantadine and rimantadine) and two neuroaminidase (NA) inhibitors (oseltamivir and zanamivir).

Amantadine and rimantadine constituted the first generation of influenza antiviral drugs. At low concentrations, amantadine and rimantadine specifically inhibit the ion-channel activity of the M2 protein [6]. The use of M2 inhibitors has been limited by the emergence of drug-resistant strains of influenza viruses. Although resistant viruses were previously uncommon [7], they have now become more frequent. Indeed, in a recent study, Bright et al. observed that, over a decade (1994-2004) of surveillance, drug resistance significantly increased: from 0.4% in 1994–1995 to 12.3% in 2003–2004 [8].

The WHO has declared oseltamivir phosphate to be the main anti-influenza medication [9]. However, oseltamivir has been linked to a surprisingly high frequency of drug-resistant strains in children [10].

It is therefore very important to develop alternative molecules for the prevention and treatment of influenza [11]. Indeed, other antiviral compounds, such as peramivir and lanamivir, are now under development and many molecules, such as chloroquine, cyanomivir, clarithromycin (CAM), nitric oxide and small interfering RNA (siRNA), have proved to be active against influenza infection *in vitro* and/or in animal models [12-16].

The combination of two antivirals or of antivirals with immunomodulators (for instance with polyriboinosinic polyribocytidylic acid [Poly ICLC] and Interferon-alpha) [17] has also been suggested, while Thymosin-alpha 1 has been shown to potentiate the immune-response elicited by contemporary vaccination in humans [18].

Drugs such as omeprazole, lansoprazole and pantoprazole selectively and irreversibly inhibit the part of the “proton pump” that performs the final step in the acid secretory process [19]. In 2005, Sasaki et al. demonstrated an anti-Rhinovirus activity of lansoprazole, which was probably due to an endosomal anti-acidic mechanism [20].

The mechanism of endosomal acidification also plays a crucial role in the replication of influenza viruses. Indeed, after the influenza virus is coupled to the cellular receptor, the virus-receptor complex is incorporated into the cytoplasm through the mechanism of endocytosis. As the endosome moves towards the nucleus, its pH decreases. This change is takes place through a cellular channel that pumps protons (H+) into the endosome. When, the pH inside the endosome reaches 5.0, hemagglutinin undergoes a structural rearrangement. This change allows the exposure of a short peptide that enables the viral envelope to fuse with the membrane of the endosome. When this happens, the viral nucleic acid is released into the cytoplasm. The nucleic acid is then transported to the nucleus of the cell. However, the RNA cannot enter the nucleus, as this latter is surrounded by the capsid proteins of the shell, such as the M1 protein. However, at the level of the envelope, the virus has an abundance of M2 protein, which creates a channel that actively pumps protons from the endosome into the virion; the lowering of the pH within the capsid enables the M1 protein to detach from the viral RNA, which, once free, can enter the cell nucleus, where replication can occur [21]. Therefore, hypothetically, by hindering the M2 ion channel, omeprazole and its derivatives could be useful in the prevention and/or therapy of Influenza-like Illness (ILI).

In order to evaluate the hypothesized protective action of Omeprazole Family Compounds (OFC) against Influenza-like Illness, a matched case-control study was performed. We chose to conduct this type of study because, in comparison with other study designs, a case-control study can yield important scientific findings at relatively little cost in terms of time, money and effort [22]. Furthermore, it provides a basis on which prospective studies can be planned.

## Methods

The Ethics Committee of S. Martino Hospital (Genoa, Italy) approved the study protocol and the written informed consent form (N° 17/2010).

### Study design

A matched case-control study was performed during the 2010-11 influenza season in Genoa (Italy). Both cases and controls were recruited by 4 General Practitioners (GPs), each of whom has about 1,200 patients aged over 18 years. Cases and controls were matched in a 1:1 ratio on the basis of gender, age (+/- 3 years) and socio-economic status (evaluated on the basis of educational level and the district of residence). Each case and matched control had the same GP.

### Case definition and selection

The potential cases were all subjects who had had at least one episode of ILI during the study period (December 2010-March 2011). Only subjects who communicated the disease to their GP were recruited. The case-definition of ILI was: presence of fever >38°C (100.4°F) and at least one other systemic symptom (headache, malaise, myalgia, chills or sweats, retrosternal pain, asthenia) and at least one respiratory symptom (cough, sore throat, nasal congestion or runny nose) during the study period [23,24].

The exclusion criteria for cases were: refusal to participate in the study and inability to provide informed consent.

### Control definition and selection

The controls were all subjects who had not had ILI during the study period (December 2010-March 2011).

At the moment of recruitment of each case, GP identified potential control subjects corresponding to matching criteria from among the patients registered in his databases. At the end of the study period (31 March 2011) controls were entered into the study by randomly selecting each control from within the group of potential candidates identified at the time of recruitment of the cases. Each GP then contacted the chosen control by telephone in order to ascertain that he/she had not had ILI during the study period. To minimize the possibility of enlisting a false negative control, the GP read the definition of ILI and provided any information necessary. In the event of refusal to participate (no signed informed consent), the GP excluded the subject and contacted the next subject on the list of potential controls.

### Study period (recruitment)

The diagnosis of ILI was based on clinical definition. In order to improve the specificity of diagnoses, the study period was therefore limited to the weeks of the influenza peak (December 2010-March 2011) according to the Italian Influenza Surveillance Network (INFLUNET) [25].

### Data collection

All GPs and researchers who carried out the study strictly complied with Italian regulations on privacy [26]. GPs administered an *ad hoc* written questionnaire to both cases and controls. The questionnaire was administered either by telephone or by face-to-face interview, after written informed consent had been obtained. The following data were recorded: personal habits (smoking, alcohol consumption [more than 2 glasses of wine or more than one glass of spirits a day]) and the presence of underlying diseases, which were classified in accordance with the International Statistical Classification of Diseases and Related Health Problems (ICD-10) [27]. Specifically, cardiovascular diseases (ICD-10 codes: I00-I02; I05-I09; I20-I99), hypertension (ICD-10 codes: I10 – I15), respiratory diseases (ICD-10 codes: J00 – J99 excluding J09 – J18), kidney diseases (ICD-10 codes: N00 – N99), diabetes (ICD-10 codes: E10-E14), cancer (ICD-10 codes: C00-D48), dyslipidemia (ICD-10 code: E78), gastric ulcer (ICD-10 code: K-25) and gastric diseases (ICD-10 codes K-21; K28-K31) were recorded. Furthermore, GPs collected data on influenza vaccination and therapy with OFC (omeprazole, esomeprazole, lansoprazole, pantoprazole, rabeprazole), fibrates (bezafibrate, ciprofibrate, clofibrate, gemfibrozil, etc), statins (atorvastatin, fluvastatin, lovastatin, simvastatin, etc) and antibiotics. OFC were administered at a dosage of 20-40 mg/day for a period of at least 2 weeks before enrolment, and this therapy was continued for at least 8 weeks. With regard to fibrates, these had to be assumed by at least 30 days before the moment of enrolment; Gemfibrozil, for instance, was administered at a dosage of between 900 and 1200 mg/day, preferably 1200 mg, and was continued for long periods of time. Finally, with regard to statins, these also had to be assumed by at least 30 days before the moment of enrolment. As an example of the dosage of statins, atorvastatin was administered at dosages of 10-80 mg/day for long periods.

All data collected from questionnaires regarding the anamnesis, therapy and vaccination status were verified by patients’ electronic records.

### Statistical analysis

The characteristics of the study population are reported as means and standard deviation (SD) for continuous variables and as proportions for categorical variables. Differences between cases and controls were analysed by means of McNemar’s test for matched case-control studies (two-tailed *p*-value).

A multivariable conditional logistic regression model was used to evaluate the effectiveness of OFC use preliminarily and to estimate the adjusted odds ratio and its 95% Confidence Interval (CI). In the model, the binary variables “ILI” and “OFC” were designated as the “outcome” and “main exposure”, respectively. The decision regarding which factors to include in the multivariable model was based on a conceptual framework describing the hierarchical relationships [28] between exposures (Table 1), with the interaction term (OFC & Influenza vaccination) being entered at a separate level. The results of the interaction analyses are presented both as the separate effects of the two exposures and as their joint effects (Table 2). In accordance with the general consensus in the epidemiological community [29,30], we presented the interaction effects on the additive scale, as this approach is the most appropriate for public health purposes. To calculate the measure of interaction, the two exposures “OFC” and “Influenza vaccination”, as preventive factors, were recoded “in such a way that the stratum with the lowest risk, when both factors are considered jointly, becomes the reference category” [31]. The measure of interaction RERI (Relative Excess Risk due to Interaction) was then calculated (Table 2) in order to examine the presence of interaction by using measurements derived from the logistic regression [31-34]. In order to estimate all three ORs that are needed to assess biological interaction, the model was set up in such a way as to include terms for three of the four possible combinations of exposure, while the fourth category served as a reference category.

**Table 1:** Hierarchical theoretical model of potential proactive action of the omeprazole family compounds against influenza viruses

***First Level***		
**Demographic characteristics**	**Lifestyle habits**	**Underlying Pathologies**
Aged 65 +	Smoking	Cardiovascular diseases
	Drinking	Hypertension
		Respiratory diseases
		Diabetes
		Cancer
		Kidney diseases
		Dyslipidaemia
		Gastric ulcer
		Gastric diseases
***Second Level***		
**Drugs taken**		
Fibrates		
Statins		
Antibiotics		
Omeprazole Family Compounds (OFC)		
***Third Level***		
**Vaccination status**		
Influenza vaccination		
***Outcome***		
Influenza-Like Illness (ILI)		

**Table 2:** Separate and joint effects of the preventive exposures “Influenza vaccination” and “OFC” on the risk of ILI after recoding and results of analyses of interaction

**Odds Ratios representing separate effects**	**OR (95% CI)**
Non-OFC users	4.38 (2.54 – 7.53)
Users of OFC	1 (reference category)
Not Vaccinated	3.8 (2.15 – 6.71)
Vaccinated	1 (reference category)
**Odds Ratios representing joint effects**	
OR11 non-OFC users and unvaccinated	5.75 (2.71 – 12.22)*
OR10 non-OFC users but vaccinated	1.48 (0.64 – 3.40)
OR01 users of OFC but unvaccinated	0.92 (0.33 – 2.53)
OR00 users of OFC and vaccinated	1 (reference category)
**Measure of effect of interaction on additive scale**	
RERI	4.34 (1.46 - 7.26)

*Observed OR = 5.75, while expected OR in the absence of interaction on an additive scale was 1.40, calculated as [(1.48 – 1) + (0.92 – 1) + 1], where 1.48 = OR non-OFC users but vaccinated and 0.92 = OR users of OFC but unvaccinated.

Analyses were performed by means of SPSS vers.16.0 for Windows, EpiInfo vers. 3.5.3, Graph-Pad software and an Excel sheet available at: http://www.epinet.se for the assessment of biological interaction. The coefficients estimated by the conditional logistic regression were obtained by using the procedure of SPSS COXREGR; this is equivalent to the conditional logistic regression when there is only one case and one or more controls in each layer.

## Results

In the Liguria Region, influenza activity was moderate in the 2010-2011 season. The influenza epidemic began in the 50^th^ week of 2010 and lasted until the 10^th^ week of 2011. The predominant circulating influenza virus was A/California/07/09_pdm_ (67%), followed by B virus (B/Brisbane/60/2008) (23.5%) and A/H3N2 (A/Perth/16/2009) (9.3%). In the 2010-2011 season there was optimal matching between circulating viruses and vaccine strains. No drifted influenza strains were isolated in the Genoa district.

Nine cases and 12 controls refused to participate in the study.

A total of 508 subjects (256 males and 252 females) were recruited: mean age 51.4 (SD = 14.5) years (males 51.7 (SD = 14.6); females 51.1 (SD = 14.3)); median age 49; interquartile range (IR) 42-61 years; range 29-94 years. The mean age (SD) of cases and controls was 51.3 (SD = 14.5) and 51.6 (SD = 14.5), respectively.

Table 3 shows the main characteristics of the cases and controls, broken down by demographic characteristics, lifestyle habits, underlying pathologies, drug use and influenza vaccination status. Cases and controls had similar lifestyle habits. Cardiovascular diseases, dyslipidaemia and gastric ulcers were significantly more common among controls than among cases. Furthermore, more controls than cases took fibrates (*p* = 0.046) and statins (*p* < 0.0001). Regarding OFC use, these drugs were taken by 7.5% of cases and 28.7% of controls (*p* < 0.0001). Only 25.6% of the subjects studied had received the influenza vaccine, the frequency of vaccination being significantly higher among controls (44/254 cases, 17.3%; 86/254 controls, 33.9%, *p* < 0.0001).

**Table 3:** Main characteristics of cases and controls, broken down by demographic features, lifestyle habits, underlying pathologies, drug use and influenza vaccination status

**VARIABLES**	**Cases (n = 254)**	**Controls (n = 254)**	*p*-value^*^
**Demographic features and lifestyle habits**			
Aged 65 yrs or more^^^	53 (20.9%)	50 (19.7%)	0.45
Smoking^§^	37 (14.6%)	45 (17.7%)	0.40
Drinking^†§^	23 (9.1%)	13 (5.1%)	0.12
**Underlying Pathologies**			
Cardiovascular diseases^§^	14 (5.5%)	38 (15.0%)	**0.0004**
Hypertension^§^	56 (22.0%)	53 (20.9%)	0.80
Respiratory diseases^§^	21 (8.3%)	21 (8.3%)	0.87
Diabetes^§^	9 (3.5%)	19 (7.5%)	0.06
Cancer^§^	3 (1.2%)	5 (2.0%)	0.72
Kidney diseases^§^	7 (2.8%)	8 (3.1%)	1.00
Dyslipidemia^§^	9 (3.5%)	45 (17.7%)	**<0.0001**
Gastric ulcer^§^	5 (2.0%)	29 (11.4%)	**<0.0001**
Gastric diseases^§ ǂ^	5 (2.0%)	14 (5.5%)	0.07
**Drugs taken**			
Fibrates^§^	1 (0.4%)	8 (3.1%)	0.046^**^
Statins^§^	9 (3.5%)	44 (17.3%)	**<0.0001**
Antibiotics^§^	8 (3.1%)	5 (2.0%)	0.58
Omeprazole family compounds^§^	19 (7.5%)	73 (28.7%)	**<0.0001**
**Vaccination status**			
Influenza vaccination^§^	44 (17.3%)	86 (33.9%)	**<0.0001**

^^^Reference: Less than 65 yrs.

^†^Reference: More than 2 glasses of wine or more than one glass of spirits a day.

^§^Reference: NO.

^ǂ^Except Gastric ulcer.

*Two-tailed *p*-value (McNemar’s test for matched case-control studies).

**According to conventional criteria, this difference is considered to be statistically significant.

The statistical significant p values are reported in bold.

Table 4 shows the results of the multivariable analysis (hierarchical approach), which was carried out in order to examine the overall contribution of demographic and lifestyle variables and underlying pathologies (Level 1), drug use (Level 2) and influenza vaccination status (Level 3) and the interactive effects of OFC and influenza vaccination in predicting ILI.

**Table 4:** Odds Ratios for ILI occurrence in the study population in the 2010/11 influenza season as a function of demographic features, lifestyle, underlying diseases and drug use, n = 508 (Multivariable analysis – Hierarchical approach)

	**Multivariable analysis**								
**Variables**	**Adjusted OR**^**§**^	**95% CI**	***p***	**Adjusted OR**^**§**^	**95% CI**	*** p***	**Adjusted OR**^**§**^	**95% CI**	*** p***
	***Level 1***			***Level 2***			***Level 3***		
**Demographic features and lifestyle habits**									
Aged 65 yrs or more*	1.94	0.24;15.6	0.53						
Smoking**	0.64	0.37;1.14	0.13						
Drinking**^#^	3.04	1.22;7.59	**0.02**	2.17					0.87;5.51	**0.09**^**^**^	2.10	0.87;4.94	0.10
**Underlying pathologies**													
Cardiovascular diseases**	0.34	0.14;0.83	**0.02**	0.56	0.22;1.39	0.21							
Hypertension**	1.26	0.68;2.35	0.47										
Respiratory diseases**	1.64	0.73;3.71	0.23										
Diabetes**	0.96	0.29;3.17	0.94										
Cancer**	1.08	0.15;7.93	0.94										
Kidney diseases**	0.61	0.14;2.71	0.51										
Dyslipidaemia**	0.17	0.07;0.42	**0.0001**	0.12	1.56	0.20							
Gastric ulcer**	0.20	0.07;0.54	**0.002**	1.18	0.31;4.57	0.82							
Gastric diseases**°	0.38	0.13;1.12	**0.08**^**^**^	1.18	0.30;4.57	0.81							
**Drugs taken**													
Fibrates**				0.08	0.05;1.41	**0.09**^**^**^	0.03	0.01;0.50	**0.02**				
Statins**				0.28	0.07;1.15	**0.08**^**^**^	0.15	0.05;0.43	**<0.001**				
Antibiotics**				1.75	0.53;5.79	0.36							
Omeprazole family compounds**				0.31	0.13;0.76	**0.01**	0.29	0.15;0.52	**<0.001**				
**Vaccination status**													
Influenza vaccination**							0.31	0.16;0.58	**<0.001**				

^§^Conditional logistic regression models.

^^^As several variables were included in each level, only those variables reaching p < 0.1 were kept in the next step, in order to avoid unstable estimates in subsequent models.

^*^Reference: *Less than 65 yrs.*

^**^Reference: *NO.*

^#^Reference: *More than 2 glasses of wine or more than one glass of spirits a day.*

°Except Gastric ulcer.

The statistical significant p values are reported in bold.

The use of OFC was associated with a lower probability of ILI (OR_adj_ = 0.31; 95% CI: 0.13-0.76), after controlling for demographic and lifestyle variables, underlying pathologies and the use of other drugs (*p* < 0.001; Level 2). The association was very similar after adjustment for influenza vaccination status (OFC OR_adj_ = 0.29; 95% CI: 0.15-0.52, Level 3).

The results of the analyses of the interaction between Omeprazole compounds (exposure of interest) and Influenza Vaccination on the risk of ILI are showed in Table 2, which reports the separate effect of each exposure and their joint effects; this approach enables the presence of interaction to be evaluated on both an additive and a multiplicative scale. A measure of interaction on the additive scale, RERI (Relative Excess Risk due to Interaction), was used to assess whether there was synergism between the two exposures. RERI >0 implies such synergism. In our analysis, we found RERI = 4.34; this means that, owing to the presence of interaction, the ILI risk for unvaccinated non-OFC users was higher than would be expected in the absence of interaction on an additive scale (observed: OR = 5.75 *vs.* expected: OR = 1.40, obtained by OR for non-OFC users but vaccinated and OR for users of OFC but unvaccinated, see Table 2), and that the test for interaction was statistically significant (RERI = 4.34; 95% CI: 1.46–7.26). Thus, there were interesting indications of a joint effect, but this needs to be confirmed in future ad hoc studies.

## Discussion

Although influenza vaccines provide a “suboptimal” rate of protection, they are the best means of avoiding influenza infection. However, in terms of both prevention and treatment, antivirals could be a complementary means of fighting the disease and mitigating its consequences. For this reason, numerous studies have been aimed at obtaining efficacious antiviral molecules. In particular, compounds of the amantadane group and the anti-neurominidase drugs have been studied and licensed for human use.

Moreover, other types of treatment are being sought. An innovative approach might be to use omeprazole family compounds as a preventive measure or as a curative treatment for influenza disease. Because of the mechanism of action of these molecules, their effect on rhinoviruses and other types of viral infection has been studied [20,35].

The results of the present study seem to confirm our hypothesis that treatment with omeprazole or similar molecules can prevent ILI. Indeed, on the basis of the results of multivariable conditional logistic regression, after control for potential confounders, the subjects treated with omeprazole family drugs displayed a lower risk of catching ILI. Specifically, this risk (OR_adj_ = 0.29, 95% CI: 0.15-0.52) was about two-thirds lower than that of subjects not treated with OFC. We also evaluated the joint effects of the absence of influenza vaccination and OFC treatment, and found that the risk of ILI in unvaccinated non-OFC users was about six times than that in vaccinated OFC users (Table 2).

Our study appears to confirm the hypothesis that, by blockading the acidification of the cell environment, the omeprazole family compounds may interfere with the mechanism of fusion of the virus envelope and endosomal membrane. More specifically, it is possible tentatively to offer an explanation of the mechanism of action of OFC in protecting against influenza infection. Indeed, OFC specifically and irreversibly inhibit the H+/K + -ATPase proton pump. Furthermore, like other molecules, such as N-ethylmaleimide (NEM), OFC inhibit ATP synthase, thus modifying Cys residues [36]. This would result in the block of the proton pump in a centripetal direction, too. Consequently, the mechanism of acidification may be inhibited in different cellular compartments, including mitochondria and endosomes. All this could reduce the efficiency of the M2 proton pump of influenza viruses, which is mediated by a small integral membrane protein (A/M2 and B/M2 for influenza A and B viruses, respectively) [37]. Our hypothesis is supported by the results obtained by Sasaki et al. (2005), who studied the inhibition of Rhinovirus infection in cultured human tracheal epithelial cells treated with lansoprazole. The entry of RNA of type 14 Rhinovirus into the cytoplasm of infected cells is thought to be mediated by the destabilization of receptor binding and by endosomal acidification [20]. Sasaki et al. found that lansoprazole reduced the fluorescence intensity of acidic endosomes in the cells and decreased the titres of Rhinoviruses and their RNA in the supernatant cell culture medium [20]. Moreover, the drug reduced cytokine concentrations, including that of interleukin-1beta, and consequently their proinflammatory effect. Finally, lansoprazole has been seen to reduce the expression of the intercellular Rhinovirus adhesion molecule-1 (ICAM-1) [38].

Nevertheless, our results are in partial contrast with those obtained by Laheij et al. [39], who found an increased risk of pneumonia in patients treated with omeprazole family molecules. However, the authors themselves admitted that various types of bias could have influenced their results, such as errors of classification (e.g. mild cases of pneumonia were neither confirmed radiologically nor microbiologically) or uncertainties regarding exposure to the drug. Finally, Laheij et al. [39] commented that the increased risk of pneumonia was very probably related to an increased susceptibility to bacterial infections, with colonization of the oral space occurring via the stomach.

Regarding underlying pathologies, our analysis showed that respiratory and cardiovascular diseases were not associated with a higher risk of catching ILI (Table 4), whereas it is well known that these underlying pathologies are associated with a higher risk of hospitalization for influenza and pneumonia. The fact that respiratory and cardiovascular diseases were more frequent in controls than in cases can probably be explained by the fact that patients with these disorders are among those for whom influenza vaccination is particularly recommended.

Furthermore, dyslipidemia and gastric diseases seemed to reduce the risk of ILI. The possible explanation for this “paradox” could be that subjects with gastric diseases regularly take OFC, and these drugs displayed a protective effect against ILI. In addition, subjects with dyslipidemia regularly take statins or fibrates, which displayed potential protection against ILI (OR_adj_ 0.15; 95% CI: 0.05-0.43). This finding is consistent with the results of other authors, who have found a protective role of these molecules (statins) with regard to hospitalization for influenza complications, such as pneumonia, in diabetic patients [40]. However, we almost always considered only uncomplicated cases of ILI.

Regarding the limitations of present study, it is well known that case-control studies are susceptible to various types of bias. Although we tried to minimize any such biases, it is possible that there may have been misclassifications regarding the diagnosis of ILI in both cases and controls. However, in order to minimize this possibility with regard to cases, we performed our study during the peak influenza period, and the criteria of case definition were rigorously applied by GPs. Misclassification among controls was minimized by rigorously explaining the definition of ILI to the subjects. Moreover, the risk of information bias regarding drug use was low, as the GPs scrupulously recorded their patients’ therapies in electronic files.

In addition, in order to minimize any selection bias, controls were matched to cases on the basis of age, sex and socio-economic status. Indeed, controls and cases were randomly selected from among the same GPs’ lists. Consequently, as they lived in the same area of the city, their socio-economic status was fairly similar.

Finally, to minimize the possibility of any confounding bias, we considered a large number of variables, such as the presence of underlying diseases, and analysed our data by means of multivariate conditional analysis.

## Conclusions

Although further confirmation is necessary, it may be concluded that omeprazole molecules could be profitably used in the prevention and, perhaps, treatment of ILI. This latter application could be particularly useful in complicated forms of the disease, the treatment of which might also involve combination with other antiviral and/or antibiotic drugs.

Our findings could prompt the planning of clinical trials aimed at establishing the best protocol of preventive or/and therapeutic treatment with OFC (dosage, timing, contraindications, side effects, etc.).

To confirm these results, our research group has planned a prospective study for the 2013-2014 influenza season, possibly involving viral confirmation of influenza in the laboratory.

## Abbreviations

ILI: Influenza-like Illness; OR: Odds Ratio; OR_adj_: Odds Ratio adjusted; CI: Confidence Interval; WHO: World Health Organization; NA: Neuroaminidase; CAM: Clarithromycin; siRNA: Small interfering RNA; Poly ICLC: Polyriboinosinic polyribocytidylic acid; OFC: Omeprazole Family Compounds; GPs: General Practitioners; INFLUNET: Italian Influenza Surveillance Network; SD: Standard Deviation; RERI: Relative Excess Risk due to Interaction; NEM: N-ethylmaleimide.

## Competing interests

The authors declare that they have no competing interests.

## Authors’ contributions

RG conceived, designed and coordinated the research. PLL, FC, CT, SB, MLC and DA collected data. PLL, FC, CT SB and MLC performed the data quality control. PLL and SR optimized the informatics database. RG, PLL, SR and DP performed the statistical analyses. RG, PLL, SR, DA and DP evaluated the results. RG, DA, SR and DP wrote the manuscript. All Authors revised the manuscript and gave their contribution to improve the paper. All authors read and approved the final manuscript.

## Pre-publication history

The pre-publication history for this paper can be accessed here:

http://www.biomedcentral.com/1471-2334/14/297/prepub
